# Prismatic treatment of acute acquired concomitant esotropia of 25 prism diopters or less

**DOI:** 10.1186/s12886-022-02501-z

**Published:** 2022-06-24

**Authors:** Yan Wu, Xueliang Feng, Junhong Li, Min Chang, Jingjing Wang, Hua Yan

**Affiliations:** 1 Department of Ophthalmology, Tianjin Medical University General Hospital, No. 154, Anshan Road, Tianjin, 300052 China; 2 Department of Strabismus and Pediatric Ophthalmology, Shanxi Eye Hospital, Taiyuan, 030002 Shanxi China; 3 Department of Medical Services, Shanxi Eye Hospital, Taiyuan, 030002 Shanxi China

**Keywords:** Acute acquired concomitant esotropia, Prismatic treatment, Stereopsis, Binocular function

## Abstract

**Background:**

This study aimed to assess the efficacy of prismatic treatment in a step-by-step manner to reduce prismatic strength in acute acquired concomitant esotropia (AACE) of 25 prism diopters (PD) or less.

**Methods:**

In this retrospective comparative study, 36 patients with AACE with deviation angle ≤ 25 PD were treated with Fresnel prism in a step-by-step manner to reduce prismatic strength. The patients were divided into two groups according to whether they regained orthophoria and were weaned off the press-on prisms within 1 year: (1) the treatment-success group, which consisted of patients who had their esotropia eliminated and were weaned off the press-on prisms within 1 year after prism correction, and (2) the treatment-continuing group, which comprised patients who needed to continue wearing a Fresnel prism at 1 year after the beginning of prismatic correction because diplopia and esotropia still existed. Clinical characteristics and cooperation were analyzed and compared between groups.

**Results:**

Fourteen of 36 patients (38.9%) were weaned off the prism and regained orthophoria and binocular single vision within 1 year after prismatic treatment. Compared with the treatment-continuing group, the treatment-success group showed smaller deviation at near and distant fixations (*P* = 0.024 and *P* = 0.006, respectively) measured at the beginning of prismatic correction, a shorter time from onset to prismatic treatment (*P* = 0.02), and a greater percentage of patients exhibiting good cooperation (*P* < 0.001).

**Conclusions:**

Prismatic treatment in a step-by-step manner to reduce prismatic strength can lead to good outcomes of motor alignment and binocular function in patients with AACE of 25 PD or less. Patients showing good cooperation, smaller angle of esotropia, and shorter duration from onset to treatment tend to eliminate esotropia and be weaned off press-on prisms within 1 year after prismatic correction.

## Background

Acute acquired concomitant esotropia (AACE) is characterized by a sudden onset of concomitant esotropia with diplopia in older children and adults [1–3]. Although AACE is rare, its occurrence has been increasing in recent years; this phenomenon has been associated with excessive near work use of smartphones and other screens [4–6].

Treatment options for AACE include strabismus surgery, botulinum toxin injection into the medial rectus, and prism treatment [7–11]. However, surgery has some drawbacks, including surgical trauma and postponed performance until 6 months after onset, resulting in a potentially prolonged period of binocular vision interruption [12, 13]. Complications of botulinum toxin injection, such as transient postoperative ptosis and exotropia, are not tolerable in some patients. In contrast, prismatic treatment is atraumatic and free of complications, and base-out prisms are often prescribed for small-angle AACE. However, to the best of our knowledge, prismatic treatment for AACE has been administered only to resolve diplopia and regain fusion without correction of abnormal alignment. Moreover, no study in the literature has showed esodeviation in patients with AACE can be corrected with prismatic treatment alone.

Some studies have shown that in patients with consecutive esotropia or partially accommodative esotropia, the use of base-out prisms regained binocular fusion, stimulated fusional divergence, and achieved orthophoria in a step-by-step manner to reduce prismatic strength [14–17].

In the present study, we designed a prismatic correction for patients with AACE with a small-angle deviation ≤ 25 prism diopters (PD) that allows for esotropia elimination and prism weaning in a step-by-step manner. After refractive correction, a base-out Fresnel prism was prescribed to each patient with small-angle esotropia.

This study assessed the efficacy of prismatic treatment in a step-by-step manner to reduce prismatic strength in small-angle (≤ 25 PD) AACE.

## Methods

### Patients

This retrospective study included 36 patients with AACE with a deviation angle ≤ 25 PD diagnosed between October 2018 and December 2020 at the Department of Strabismus and Pediatric Ophthalmology of Shanxi Eye Hospital. This study adhered to the tenets of the Declaration of Helsinki and was approved by the Ethics Committee of Shanxi Eye Hospital (approval number SXYYLL-20200627). Written informed consent for the use of clinical records in this study was obtained from all participants and their guardians.

Patients who met the following criteria were included: patients with acute-onset acquired concomitant esotropia with diplopia; patients with best-corrected visual acuity of 20/20 or better in both eyes; and patients with deviation angle ≤ 25 PD and deviation in all directions of gaze differing by < 2 PD.

The exclusion criteria were as follows: patients with a reduction of 10 PD or more in the esodeviation after full hypermetropic spectacle correction; patients with a history of ophthalmic deficits, including amblyopia, strabismus, or other eye diseases; patients with a history of eye surgery; patients with systemic disease and neurological disease confirmed by systemic neurological examinations; and patients with “sagging eye” syndrome.

### Ophthalmological examination

The patients’ medical and near work histories were carefully assessed. The data collected included sex, age, visual acuity, time from onset to treatment, cycloplegic refractive error, Worth 4-dot test score, angle of deviation, and measurement of stereopsis with Titmus stereotest. The same optometrist measured the uncorrected and best-corrected visual acuity. The basic ophthalmological examination included anterior segment evaluation using slit-lamp microscopy and ophthalmoscopy. Orthoptic examinations, including assessment of ocular movements, angle of deviation, and sensory status, were performed by a single strabismologist. The strabismus angle was measured with the alternate prism cover test with accommodative targets for fixation at 0.33 m and 6 m, with full refractive correction. Binocular functions (sensory status) were evaluated using the Titmus stereotest (circles test) and the Worth 4-dot test at 6 m and 0.33 m. In the Titmus stereotest, stereopsis was stratified into three levels: ≤ 60 s of arc, 80–800 s of arc, and > 800 s of arc. A result of 60 s of arc or less was recorded as normal stereoacuity. However, a result of 80–800 s of arc was recorded as abnormal stereoacuity. The result was recorded as negative when the patient could not distinguish any circular stereopsis (> 800 s of arc). In the Worth 4-dot test, only a fusional response of four light visible was considered normal. Refractive errors of patients aged ≤ 14 years were determined using cycloplegic refraction performed after 1% atropine eye drops instillation, and those of patients aged > 14 years were determined using 1% tropicamide. Subsequently, the refractive errors were analyzed as spherical equivalent values. Before prismatic treatment, all patients underwent brain and orbital magnetic resonance imaging (MRI) scans and systemic neurological examinations. Serological tests including blood glucose, blood routine, thyroid function test and anti-thyroid related antibodies were performed to exclude systemic diseases related to strabismus.

### Treatment methods

After refractive correction, base-out Fresnel press-on prisms, 2 to 10 PD less than the angle of deviation, were prescribed to all patients and applied only to the bottom of a spectacle lens. The specific amount of prisms prescribed for each patient depended on diplopia elimination at near fixation. Slight diplopia could still be present when patients looked at distant objects in a relaxed state, whereas it could be eliminated when striving to stimulate the divergence power. Therefore, it was necessary to encourage the patients to look at a distant target for as long as possible to eliminate slight diplopia and stimulate the divergence power. When the strength of press-on prism is ≤ 10 PD, the prism is pressed on the refractive lens of the dominant eye; when the strength of the prism is > 10 PD, the prisms were distributed onto the lenses of both eyes. Follow-up examinations were performed monthly until the 1 year after the prescription of prism glasses. The angle of deviation, stereoacuity, and prismatic treatment results were evaluated at follow-up.

To maintain fusion, changing the strength of the press-on prisms once or several times is necessary when the angle of deviation changes. If there was a reduction in esotropia on follow-up examination after at least 1 month, the strength of the prisms was accordingly reduced, still maintaining 2–10 PD less than the angle of deviation, and weaning of prisms was recommended when a patient regained orthophoria and fusion without prisms.

### Group classification

The patients were divided into the following two groups according to whether they regained orthophoria and were weaned off the press-on prisms within 1 year: (1) the treatment-success group, which consisted of patients who had their esotropia eliminated and were weaned off the press-on prisms within 1 year after prism correction, and (2) the treatment-continuing group, which comprised patients who needed to continue wearing a Fresnel prism at 1 year after the beginning of prismatic correction because diplopia and esotropia still existed.

### Treatment cooperation measurement

Good cooperation was assessed based on the following: wearing of prism glasses always and looking at a distant target for at least 2 h daily and more than 20 min at a time. If the above standards cannot be met, cooperation is considered poor.

### Main outcome measures

The main outcome measures were the angle of deviation and sensory status at 1 year after prismatic correction. Success was defined as regaining orthophoria (deviation of 3 PD or less at both near and distance fixations) with evidence of binocular single vision when prisms were weaned off. Binocular single vision was defined as the absence of diplopia with a fusional response in the Worth 4-dot test (four light visible for both near and distance fixations).

### Statistical analyses

Statistical analyses were performed using SPSS version 26.0 (IBM Corp., Armonk, NY, USA). The Shapiro–Wilk test was used to assess data normality. Fisher’s exact test, Student’s t-test (independent-samples t-test), and the Wilcoxon rank-sum test were used to compare characteristics at the beginning of prism correction between the treatment-success and treatment-continuing groups. Clinical factors for the success of prismatic treatment were then introduced into a binary logistic regression model, and to find the best multivariate model, the stepwise procedures was used. Relative clinical factors were estimated as odds ratios (OR) with a 95% confidence interval (CI). Stereoacuity was compared between the beginning and 1 year after prismatic correction in the two groups using the Wilcoxon rank-sum test. The change in the angle of deviation at the beginning of prismatic correction and 1 year later in the treatment-continuing was compared using the Wilcoxon rank-sum test. Statistical significance was set at *P* < 0.05.

## Results

### Characteristics of the patients

A total of 40 patients with AACE met the inclusion criteria, but 4 patients who underwent strabismus surgery were excluded. Among this 4 patients, 2 patients tried prismatic correction before strabismus surgery and later abandoned treatment less than 6 months after prismatic treatment due to the cosmetic problems made by the Fresnel prism, and the other 2 patients wanted to have their esodeviation corrected immediately, so they initially chose strabismus surgery.

In total, 36 patients were included in this study, including 16 males and 20 females. The mean age (± standard deviation) of 36 patients at the beginning of prismatic correction was 28.9 ± 11.7 (range, 11–60) years. The mean time from AACE onset to treatment was 12.1 ± 10.3 (range, 1–36) months. Brain and orbital MRI scans yielded normal findings for all patients. One, two, and 33 patients had emmetropia, mild hyperopia, and myopia with a spherical equivalent of − 4.6 ± 1.7 diopters (D) in the right eye and − 4.5 ± 1.7 D in the left eye (range, − 0.25 D to − 8.75 D), respectively. All patients had a history of near work use of smartphones and/or other screens for a median of 6 (range, 5–10) h each day.

### Angles of esotropia and sensory status at the time of prescription of the Fresnel prisms

The median esodeviations at distant and near fixations were 15.00 (range, 5–25) PD and 12.50 (range, 5–25) PD, respectively. For 31 (86.1%) patients, the angles of deviation for distant and near fixations were equal (differing by ≤  ± 5 PD), whereas for the remaining five (13.9%) patients, deviations at distant and near fixations were unequal (differing by >  ± 5 PD). Titmus stereoacuity ranged from 40 to > 800 s of arc. Five (13.9%) patients had no stereopsis (> 800 s of arc, negative stereopsis), 13 (36.1%) had stereopsis of 80–800 s of arc, and 18 (50.0%) had ≤ 60 s of arc. In the Worth 4-dot test, all patients showed five light visible for distant fixation. Moreover, three patients showed four light visible, whereas the remaining 33 patients showed five light visible for near fixation.

### Efficacy of prismatic treatment

Fourteen of 36 patients (38.9%) were weaned off the prism and regained orthophoria and binocular single vision, showing four light visible for both distant and near fixations in the Worth 4-dot test within 1 year after prism prescription (the treatment-success group). Moreover, 22 (61.1%) patients were willing to continue to wear the prisms because of the still existing esotropia with diplopia at 1 year after prism prescription (the treatment-continuing group). Clinical characteristics at the beginning of prismatic treatment and patient cooperation were compared between the treatment-success and treatment-continuing groups (Table 1). The median deviation at near and distant fixations measured at the beginning of prismatic correction was significantly smaller in the treatment-success group than in the treatment-continuing group (*P* = 0.024 and *P* = 0.006, respectively). The median time from onset to prismatic treatment was significantly shorter in the treatment-success group than in the treatment-continuing group (*P* = 0.02). The percentage of patients exhibiting good cooperation was greater in the treatment-success group than in the treatment-continuing group (100% vs. 36.4%), with a statistically significant difference between the two groups (*P* < 0.001). No significant differences in sex ratio, age, cycloplegic refractive error, and stereoacuity were observed between the two groups.

**Table 1:** Clinical characteristics at the beginning of prismatic correction and cooperation of patients were compared between the treatment-success and treatment-continuing groups

Variable		Treatment-success group	Treatment-continuing group	*P*-value
No. of patients		14 (38.9%)	22 (61.1%)	
Sex ratio (male: female)		4:10	12:10	0.176^a^
Cooperation ratio (good cooperation: poor cooperation)		14:0	9:13	< 0.001^a^
Age (years)		31.50 ± 11.59	27.18 ± 11.76	0.288^b^
Median time from onset to treatment (months) [range]		4.50 [1.00–24.00]	12.00 [1.00–36.00]	0.02^c^
Spherical equivalent cycloplegic refractive error (right, diopters)		− 4.41 ± 2.89	− 4.00 ± 1.66	0.590^b^
Spherical equivalent cycloplegic refractive error (left, diopters)		− 4.25 ± 2.66	− 3.98 ± 1.76	0.713^b^
Median deviation at near fixation measured at the beginning of prism correction (PD) [range]		10.00 [5.00–20.00]	15.00 [5.00–25.00]	0.024^c^
Median deviation at distance fixation measured at the beginning of prism correction (PD) [range]		10.00 [8.00–20.00]	20.00 [5.00–25.00]	0.006^c^
Stereoacuity (s of arc)		No	No	0.529^c^
≤ 60 (normal)		8 (57.1%)	11 (50.0%)	
80–800 (abnormal)		5 (35.7%)	7 (31.8%)	
> 800 (negative)		1 (7.1%)	4 (18.2%)	

^a^Fisher’s exact test

^b^Student’s t-test

^c^Wilcoxon rank-sum test

Except for patient cooperation, the association between all other clinical characteristics above and the success of prismatic treatment was evaluated by binary logistic analysis. The patient cooperation factor was not included in the logistic analysis, because none of the patients in treatment-success group had poor cooperation (the value was zero) which leaded to abnormal OR-value. Table 2 shows the binary logistic regression model. At the stepwise-forward multivariate logistic analysis, only the deviation at distance fixation measured at the beginning of prism correction and the time from onset to treatment were significantly associated with treatment success (OR = 0.76, *P* = 0.009 and OR = 0.86, *P* = 0.017; respectively). Then a backward-stepwise approach was used, only the deviation at near fixation measured at the beginning of prism correction and the time from onset to treatment were were significantly associated with treatment success (OR = 0.72, *P* = 0.009 and OR = 0.80, *P* = 0.015; respectively). With smaller deviation at distance and near fixations measured at the beginning of prism correction and shorter time from onset to prismatic treatment, clinical success of prismatic treatment was more likely.

**Table 2:** Stepwise-forward and stepwise-backward multivariate logistic analysis on clinical factors for the success of treatment at 1 year after prismatic correction (only remaining variables)

Clinical factors	Stepwise-forward		Stepwise-backward	
	OR (95%CI)	*P*	OR (95%CI)	*P*
Deviation at distance fixation measured at the beginning of prism correction (PD)	0.76 (0.61 ~ 0.93)	0.009	-	-
Deviation at near fixation measured at the beginning of prism correction (PD)	-	-	0.72 (0.57 ~ 0.92)	0.009
Time from onset to treatment (months)	0.86 (0.76 ~ 0.97)	0.017	0.80 (0.67 ~ 0.96)	0.015
Sex		-		0.058
Female	-		0.10 (0.01 ~ 1.09)	
Male	-		1.00 (Ref)	
Spherical equivalent cycloplegic refractive error (left, diopters)	-		0.47 (0.22 ~ 1.00)	0.051

The average duration of Fresnel prism wearing in 14 patients in the treatment-success group was 6.36 ± 2.79 months. Among them, 9 patients were weaned off the press-on prisms and regained orthophoria within 6 months after prismatic treatment, while 5 patients were weaned off them and regained orthophoria during 6 months to 1 year after prismatic treatment. The 14 patients who were weaned off the prisms were followed up without prism for at least 1 year, and none of the patients experienced recurrence. The remaining 22 patients in the treatment-continuing group had a significantly smaller angle of deviation at both near and distant fixations at 1 year after prismatic correction compared with that at the beginning of prismatic correction (*P* = 0.001 and *P* < 0.001, respectively; Table 3). Titmus stereoacuity in both groups was better at 1 year after prismatic correction compared with that at the beginning of prismatic correction, with a statistically significant difference (*P* = 0.041 and *P* = 0.003, Table 4). The strabismus surgery was performed for 2 patients in the treatment-continuing group at more than 2 years after prism wearing, who failed the prismatic treatment, while the other 20 patients in the treatment-continuing group maintained prism wearing until the final visit.

**Table 3:** The angle of deviation at the beginning and at 1 year after prismatic correction in the treatment-continuing group

Variable	At the beginning	At 1 year after	*P*-value
Deviation at near fixation (PD)	15.00 [5.00–25.00]	10 [3.00–20.00]	0.001^c^
Deviation at distance fixation (PD)	20.00 [5.00–25.00]	10.00 [5.00–25.00]	< 0.001^c^

^c^Wilcoxon rank-sum test

**Table 4:** The result of Titmus stereotest at the beginning and at 1 year after prismatic correction in the two groups

	Treatment-success group (n)			Treatment-continuing group (n)		
Stereoacuity (s of arc)	≤ 60 (normal)	80–800 (abnormal)	> 800 (negative)	≤ 60 (normal)	80–800 (abnormal)	> 800 (negative)
At the beginning	8 (57.1%)	5 (35.7%)	1 (7.1%)	11 (50.0%)	7 (31.8%)	4 (18.2%)
At 1 year after	14 (100%)	0 (0.00%)	0 (0.00%)	21 (95.5%)	1 (4.5%)	0 (0.0%)
*P*-value	0.020^c^			0.004^c^		

^c^Wilcoxon rank-sum test

## Discussion

The incidence of AACE has increased in recent years and has been proven to be associated with excessive use of smartphones and other screens [4–6]. Patients with AACE experience diplopia, with variable angles of strabismus [1, 2]. AACE has been categorized into three types: (1) Swan type (caused by fusion interruption, primarily resulted by monocular vision loss or monocular obstruction), (2) Burian–Franceschetti type (often considered to be related to psychic or physical stress, involving a minimum degree of hypermetropia and a large angle of deviation), and (3) Bielschowsky type (presenting various degrees of myopia) [1, 11].

High variability in age was observed in this study, with the youngest patient being aged 11 years and the oldest 60 years, which was consistent with the age range recorded in previous studies [6, 18]. Among the 36 patients, 33 (91.7%) presented with myopia, whereas only two and one patients had mild hypermetropia and emmetropia, respectively. There is a high prevalence of myopia (80% to 90%) in the younger population in East Asia, and the incidence rate could reach ~ 95% in China [19–21], which coincided with the rate of myopia in our cases. Therefore, ethnic predisposition for myopia could partly explain why most of the 36 cases had myopia. For elderly subjects and highly myopic subjects, the degeneration of the lateral rectus-superior rectus band with inferior displacement of the lateral rectus muscle and its pulley could be a risk factor for the development of esotropia [22, 23]. In this study, all patients underwent orbital MRI to exclude degeneration and other pathological changes in the extraocular muscles and intermuscular septa. Bielschowsky [24] claimed that in patients with myopic AACE, excessive near work due to uncorrected myopia was the etiology of esotropia, which may strengthen the muscular tension of the medial rectus and break the balance between convergence and divergence. However, in this study, all patients with myopia regularly wore spectacles prior to the onset of strabismus, so we agree with other authors that sole uncorrected myopia should not be considered one of the causes of AACE [6, 25]. In this study, all patients spent extended periods (5–10 h) using smartphones and other screens that required near vision prior to AACE onset; hence, they spent more time with smartphones than the patients in Lee’ study [4]. Thus, our results are consistent with those of other studies suggesting that excessive near-vision work, which may strengthen the muscular tone of the medial rectus and break the balance between convergence and divergence, can be a risk factor for the development of esotropia [4, 5]. Prior to this study, we had observed 15 AACE patients with a deviation angle ≤ 25 PD and a history of excessive use of smartphone or other screens, treated only by reducing the use of smartphone and other screens for 3 to 9 months. However, none of these patients had the esotropia eliminated and the symptoms improved, and later, these patients chose strabismus surgery or the botulinum toxin injection. One of the important reasons for this phenomenon is that patients with AACE tend to avoid looking at the distance due to more obvious diplopia for distant targets, so that it is difficult for AACE patients to effectively reduce the time spent on near-work such as use of smartphone. So we agreed with Shi [26] that successful treatment was almost unavailable simply by recommending patients to reduce the use of smartphone and other screens. Therefore, in this study, in addition to recommending to reduce the use of smartphone and other screens for patients with AACE, we designed a nonsurgical management approach using a prism to enhance the abduction of patients, regain a balance between convergence and divergence, and achieve the effect of correcting eye position and eliminating diplopia.

Prisms are often used to resolve diplopia in patients with strabismus by altering the pathway of light, moving images onto the fovea of the deviated eye and within a range that allows fusion of the images. The thinner Fresnel prism with 1.0 mm thickness allows a wider range of prismatic corrections. In the present study, base-out Fresnel prisms were prescribed to patients and were weaned in a step-by-step manner. This approach not only uses the optical principle of the prism but also increases the abduction fusion force of patients to reduce and further eliminate esotropia. Given gradual prismatic reduction and the higher cost of treatment, ground-in prisms was not recommended even for patients with esotropia less than 14 PD, so base-out Fresnel press-on prisms were prescribed for all patients included in this study.

Several reports of prismatic treatment applied in consecutive esotropia and partially accommodative esotropia have revealed that base-out prisms may stimulate fusional divergence and eventually allow for orthophoria achievement in a step-by-step manner, reducing the prismatic strength in some patients [14, 15, 27–29]. However, some studies of prismatic correction in AACE have shown a good effect only in the resolution of diplopia but without eliminating esotropia [8, 9]. To the best of our knowledge, no previous study of prismatic correction aimed at eliminating esotropia in a step-by-step manner to reduce the prismatic strength in patients with AACE has been conducted. Given increased optical aberrations, loss of contrast, and light scatter in larger Fresnel prisms (> 12PD) [30], the subjects included in this study were patients with AACE of 25 PD or less. Besides, the prisms ≤ 10 PD were pressed on the refractive lens of the dominant eye, and the prisms > 10 PD were distributed onto the lenses of both eyes in the present srtudy.

In the present study, patients with AACE were prescribed a base-out prism 2–10 PD less than the angle of esodeviation, leaving patients with slight residual esotropia so that the patients sometimes had slight diplopia for distant fixation in a relaxed state. Ruatta [6] reported that AACE patients had wider divergent fusional amplitude for near fixation than for distant fixation, similar to that of normal subjects; therefore, there was better compensation of the deviation at near fixation, and symptoms of diplopia were more obvious when AACE patients looked at distant targets. This can also explain why the patients with under-corrected prisms in this study had diplopia eliminated at near fixation but still had slight diplopia at distant fixation in a relaxed state. To eliminate the slight diplopia, the patients would aim to mobilize the divergent fusion function to control eye position into orthophoria, simultaneously enhancing the tension of the lateral rectus, and the strength of the prism would be reduced step-by-step as the angle of deviation decreased. Therefore, in some patients, it is possible to stimulate fusional divergence, eliminate prisms, and finally regain orthophoria and fusion.

Overall (treatment-success group plus treatment-continuing group), the median angles of esodeviation at the beginning of prismatic correction were 15.00 (range, 5–25) PD for distant fixation and 12.50 (range, 5–25) PD for near fixation. In some reports on AACE, the angles of esodeviation were nearly equivalent for distant and near fixations (differing by ≤  ± 5 PD) in each patient [4, 6, 31]. In this study, the near and distant deviation measurements were equal in 86.1% (31/36) of patients but different in 13.9% (5/36) of patients (differences within 10 PD), similar to that reported by Fu Tao [18].

Fourteen out of 36 (38.9%) patients had their esotropia eliminated and were weaned off the prism glasses within 1 year after prismatic treatment (the treatment-success group). Due to the presence of diplopia and esotropia, the remaining 22 patients needed to continue wearing a Fresnel prism, and all patients were willing to wear prisms at 1 year after prismatic treatment (the treatment-continuing group). The present study revealed that patients who showed a smaller angle of deviation at the beginning of prismatic treatment and those with a shorter time from onset to treatment tended to wean off the prism within 1 year. We speculate that patients with a short time from onset to treatment are in the early stage of AACE, so the imbalance of convergence and divergence caused by the new enhancement of the medial rectus is easily reversible compared to patients with a long duration from onset to treatment. Furthermore, since all (100%) patients in the treatment-success group showed good cooperation and only 40.9% (9/22) of patients in the treatment-continuing group showed good cooperation, the results of the present study suggest that good cooperation is vitally important for successful treatment (Table 1). According to patients’ reports, all patients always wore a prism, but patients with poor cooperation could not look at distant targets for at least 2 h daily and more than 20 min at once. Thus, looking at distance target that could mobilize the divergent fusion function to eliminate slight diplopia for distant fixation is an important step in prismatic treatment in a step-by-step manner to reduce prismatic strength. In this study, on an examination performed at 1 year after prism correction, the median angles of deviation at both distant and near fixations in the remaining 22 patients in the treatment-continuing group were significantly smaller than those at the beginning of prism prescription, and stereoacuity improved among patients in both the treatment-success and treatment-continuing groups (Tables 3 and 4). Therefore, prismatic treatment in a step-by-step manner, reducing prismatic strength, may help to regain binocular fusion, reduce the angle of deviation in patients with AACE, and even cause deviations in some patients to correct to orthophoria or esophoria without surgery for esotropia or botulinum toxin injection, thus avoiding trauma and complications.

This study has some limitations. First, only patients with AACE of 25 PD or less were included; thus, the number of patients in this study was small. Second, this was a retrospective study. We observed that some patients showed evident reduction in deviation within 1 year after prism prescription; thus, 1 year was selected as the cutoff point. However, as a period of 1 year seems short, long-term observation of prism treatment performed in a step-by-step manner to reduce prismatic strength will be necessary, which may yield a different result with respect to the percentage of patients being successfully cured or with better clinical outcomes.

## Conclusions

Prismatic correction by reducing prismatic strength in a step-by-step manner can generate good outcomes of motor alignment and binocular function in patients with AACE of 25 PD or less. Patients with good cooperation, smaller angle of esotropia, and shorter duration from onset to treatment tend to eliminate esotropia and be weaned off the prism. Therefore, prismatic correction in a step-by-step manner to reduce prismatic strength allows these patients to achieve successful motor outcomes, avoiding surgical correction and botulinum toxin injection and preventing them from experiencing the trauma and complications caused by surgery and botulinum toxin injection.

## Data Availability

The datasets used and/or analyzed during the current study are available from the corresponding author upon reasonable request.

## Abbreviations

AACE: Acute acquired concomitant esotropia
PD: Prism diopter

## References

[1] Clark AC, Nelson LB, Simon JW, Wagner R, Rubin SE (1989). Acute acquired comitant esotropia. Br J Ophthalmol.

[2] Legmann Simon A, Borchert M (1997). Etiology and prognosis of acute, late-onset esotropia. Ophthalmology.

[3] Mohney BG (2007). Common forms of childhood strabismus in an incidence cohort. Am J Ophthalmol.

[4] Lee HS, Park SW, Heo H (2016). Acute acquired comitant esotropia related to excessive Smartphone use. BMC Ophthalmol.

[5] Zheng K, Han T, Han Y, Qu X (2018). Acquired distance esotropia associated with myopia in the young adult. BMC Ophthalmol.

[6] Ruatta C, Schiavi C (2020). Acute acquired concomitant esotropia associated with myopia: is the condition related to any binocular function failure. Graefes Arch Clin Exp Ophthalmol.

[7] Wang-Xue WX (2010). The role of botulinum toxin for acute-onset concomitant esotropia: a pilot study. Ophthalmol China.

[8] Burke JP, Firth AY (1995). Temporary prism treatment of acute esotropia precipitated by fusion disruption. Br J Ophthalmol.

[9] Chang F, Wang T, Yu J, Li M, Lu N, Chen X (2020). Prism treatment of acute acquired concomitant esotropia precipitated by visual confusion. Strabismus.

[10] Scott AB (1980). Botulinum toxin injection into extraocular muscles as an alternative to strabismus surgery. Ophthalmology.

[11] Dawson EL, Marshman WE, Adams GG (1999). The role of botulinum toxin A in acute-onset esotropia. Ophthalmology.

[12] Schöffler C, Sturm V (2010). Repeated surgery for acute acquired esotropia: is it worth the effort. Eur J Ophthalmol.

[13] Wan MJ, Mantagos IS, Shah AS, Kazlas M, Hunter DG (2017). Comparison of botulinum toxin with surgery for the treatment of acute-onset comitant esotropia in children. Am J Ophthalmol.

[14] Hardesty HH, Boynton JR, Keenan JP (1978). Treatment of intermittent exotropia. Arch Ophthalmol.

[15] Han SB, Hwang JM (2009). Prismatic correction of residual esotropia of 20 prism dioptres or less after full hypermetropic correction. Eye (Lond).

[16] Lee EK, Hwang JM (2013). Prismatic correction of consecutive esotropia in children after a unilateral recession and resection procedure. Ophthalmology.

[17] Choe HR, Yang HK, Hwang JM (2019). Long-term outcomes of prismatic correction in partially accommodative esotropia. PLoS One.

[18] Fu T, Wang J, Levin M, Xi P, Li D, Li J (2017). Clinical features of acute acquired comitant esotropia in the Chinese populations. Medicine (Baltimore).

[19] Holden BA, Fricke TR, Wilson DA, Jong M, Naidoo KS, Sankaridurg P (2016). Global prevalence of myopia and high myopia and temporal trends from 2000 through 2050. Ophthalmology.

[20] Jung SK, Lee JH, Kakizaki H, Jee D (2012). Prevalence of myopia and its association with body stature and educational level in 19-year-old male conscripts in seoul, South Korea. Invest Ophthalmol Vis Sci.

[21] Sun J, Zhou J, Zhao P, Lian J, Zhu H, Zhou Y (2012). High prevalence of myopia and high myopia in 5060 Chinese university students in Shanghai. Invest Ophthalmol Vis Sci.

[22] Demer JL (2008). More respect for connective tissues. J AAPOS.

[23] Kono R, Poukens V, Demer JL (2002). Quantitative analysis of the structure of the human extraocular muscle pulley system. Invest Ophthalmol Vis Sci.

[24] Das BA, der Myopen E (1922). Das Einwartsschein der Myopen. BerDtsch Ophth Gesell.

[25] Chen J, Deng D, Sun Y, Shen T, Cao G, Yan J (2015). Acute acquired concomitant esotropia: clinical features, classification, and etiology. Med (Baltim).

[26] Shi M, Zhou Y, Qin A, Cheng J, Ren H (2021). Treatment of acute acquired concomitant esotropia. BMC Ophthalmol.

[27] Hardesty HH (1968). Treatment of overcorrected intermittent exotropia. Am J Ophthalmol.

[28] Véronneau-Troutman S (1971). Fresnel prism membrane in the treatment of strabismus. Can J Ophthalmol.

[29] Kim YH, Choi MY (2006). The effect of Fresnel prism treatment in consecutive esotropia [in Korean]. J Korean Ophthalmol Soc.

[30] Gunton KB, Brown A (2012). Prism use in adult diplopia. Curr Opin Ophthalmol.

[31] Spierer A (2003). Acute concomitant esotropia of adulthood. Ophthalmology.

